# Findings from a Project Which Established Hepatitis C Point-of-Care Testing and Linkage to Care at a Homelessness Service in Adelaide, Australia, 2021–2022

**DOI:** 10.3390/v16121882

**Published:** 2024-12-04

**Authors:** Erin M. McCartney, Joshua Dawe, Lucy Ralton, Jeffrey Stewart, Jacqueline A. Richmond, Alan Wigg, Victoria Cock, Edmund Y. Tse, Tom Rees, David Shaw, Catherine Ferguson

**Affiliations:** 1 Infectious Diseases Department, Royal Adelaide Hospital, Central Adelaide Local Health Network, Adelaide 5000, Australia; lucy.ralton@sa.gov.au (L.R.); david.shaw@sa.gov.au (D.S.); catherine.ferguson@sa.gov.au (C.F.); 2 Bristol Doctoral College, Bristol Medical School, Bristol BS8 2PN, UK; joshua.dawe@bristol.ac.uk; 3 Disease Elimination, Burnet Institute, Melbourne 3004, Australia; jacqui.richmond@burnet.edu.au; 4 Infectious Disease Department, The Queen Elizabeth Hospital, Central Adelaide Local Health Network, Adelaide 5000, Australia; jeffrey.stewart@sa.gov.au; 5 Hepatology and Liver Transplantation Medicine Unit, Southern Adelaide Local Health Network, Adelaide 5042, Australia; alan.wigg@sa.gov.au; 6 Drug and Alcohol Services, Glenside 5065, Australia; victoria.cock@sa.gov.au; 7 Gastroenterology & Hepatology Department, Royal Adelaide Hospital, Adelaide 5000, Australia; edmund.tse@sa.gov.au; 8 Communicable Disease Control Branch, SA Health, Adelaide 5000, Australia; tom.rees@sa.gov.au

**Keywords:** hepatitis C virus, people who inject drugs, homelessness, point-of-care testing

## Abstract

Background: Point-of-care hepatitis C virus (HCV) testing streamlines testing and treatment pathways. In this study, we established an HCV model of care in a homelessness service by offering antibody and RNA point-of-care testing. Methods: A nurse and peer-led HCV model of care with peer support were implemented between November 2021 and April 2022 at a homelessness service in Adelaide, Australia. All clients of the service were eligible to participate. Clients were offered an initial antibody point-of-care test, and antibody positive clients were immediately offered RNA point-of-care testing. Clients who tested RNA positive were linked to a viral hepatitis nurse for treatment. Results: A total of 230 clients received an HCV antibody point-of-care test, of which 68 (30%) were antibody positive and 11 (5%) were RNA positive. Of these, seven (64%) clients successfully completed treatment and five (45%) received a sustained virological response (SVR) test to confirm cure. Conclusions: We successfully established HCV testing and a treatment pathway at a homelessness service using HCV antibody and RNA point-of-care testing. The high testing uptake underscores the utility of HCV point-of-care testing when establishing HCV testing and treatment pathways. The low RNA positivity suggests that an initial HCV antibody test was cost-effective, and the four clients diagnosed with chronic HCV who were lost to follow-up indicate a need for enhanced treatment support.

## 1. Introduction

Despite providing unrestricted access to highly curative direct acting antiviral (DAA) therapy, reductions in hepatitis C virus (HCV) diagnoses and DAA prescribing means that Australia may miss its 2030 elimination targets [1,2]. Achieving HCV elimination is therefore contingent on identifying and responding to gaps in the cascade of care, including establishing HCV testing and treatment pathways within settings servicing populations at heightened risk, such as people experiencing unstable housing and homelessness [3,4].

People who inject drugs experience social and structural risk factors such as unstable housing and homelessness which can increase their likelihood of acquiring HCV [5,6]. In Australia, around one in five people who inject drugs report recent experiences of homelessness and housing instability [6]. Experiences of homelessness are also associated with increases in injecting practices that increase the risk of HCV transmission and other drug-related harms, including sharing injecting equipment and public injecting [7,8]. People who inject drugs experiencing homelessness therefore represent a priority population for whom HCV models of care should be tailored.

Historically, HCV testing pathways have relied on multiple venipunctures to ascertain exposure to HCV and detect current infections. These pathways often require patients to attend healthcare services on multiple occasions, increasing the time between engagement and diagnosis, and the risk of loss to follow-up. HCV point-of-care testing overcomes these barriers by providing fast and reliable results. Furthermore, HCV point-of-care testing is a scalable alternative to conventional testing, meaning that testing and treatment pathways can be established in health and harm reduction services with limited clinical capacity.

The PROMPt study demonstrated a nurse and peer-led model of HCV point-of-care testing and linkage to care that was highly successful in priority settings, and indicated that HCV transmission remains a significant health issue for persons experiencing homelessness [9]. This study utilised the same study protocol as PROMPt with the aim of evaluating the outcomes of a combined nurse and peer-led HCV model of care utilising HCV point-of-care testing at a homelessness service in Adelaide, Australia. Our secondary aim was to assess the acceptability of HCV point-of-care testing among participants.

## 2. Methods

### 2.1. Intervention Design and Recruitment

Between 1 November 2021 and 11 April 2022, a nurse and peer-led HCV model of care offering point-of-care testing was implemented once per week at a homelessness service with no established HCV testing or treatment services. The homelessness service is situated in a building in the central business district of Adelaide, and provides a wide range of services, including meals, showers, laundry facilities, health services, education, legal aid, and recreation activities. The service has the capacity to provide these amenities for up to 200 clients per day.

The study team consisted of a nurse trained in viral hepatitis management and HCV peer educators. Both the nurse and peer educators administered PCOT, reported results and provided pre and post-test counselling and harm reduction education. Clients were not required to book an appointment with the study team or disclose risk factors for HCV transmission in order to participate. All clients of the service aged 18 years or older were therefore eligible. Walk-in clients of the service were offered HCV testing by either the study nurse or a peer worker.

Clients were initially offered an HCV antibody test using the SD Bioline (Abbott, Santa Clara, CA, USA) HCV antibody point-of-care fingerstick test, with results available after 5–20 min. Clients who received a positive HCV antibody test result were immediately offered an HCV RNA test using the Xpert^®^ HCV Viral Load Fingerstick assay, with results available in approximately 60 min. All point-of-care testing was conducted by a trained nurse using the GeneXpert^®^ system (Cepheid, Sunnyvale, CA, USA). Clients could either wait at the service for 60 min to receive test results, be contacted by phone by a study team member, or return for their results on the next testing day.

Clients with detectable HCV RNA were linked to care with a local viral hepatitis nurse to assess suitability for treatment, for pre-treatment counselling, and to receive a prescription for DAA treatment. Treatment support, monitoring, and SVR testing were then managed by the viral hepatitis nurse.

### 2.2. Data Collection

The demographic characteristics of clients were collected by the study nurse, and included age, gender, and whether clients identified as Aboriginal and/or Torres Strait Islander. Clients were also asked whether they had ever previously received an HCV test (antibody or RNA). To assess the acceptability of HCV point-of-care testing, clients were asked (1) “Is it important for you to get your hepatitis C result on the same days as getting tested?” and (2) “If you could choose which type of testing for hepatitis C would you prefer?”.

The results of the HCV point-of-care tests and subsequent treatment outcomes among those who tested HCV RNA positive were recorded by the study nurse. Data were collected from participants by the study nurse and entered into a database using Research Electronic Data Capture (REDCap) software, Version 10.6.28 (Vanderbilt University; https://www.project-redcap.org/ (accessed on 19 September 2024)).

### 2.3. Data Analysis

Descriptive statistics were presented for clients who received an HCV antibody point-of-care test. An HCV cascade of care was presented to describe the sequential steps through which clients were diagnosed and treated for HCV, and included HCV antibody testing, HCV RNA testing, linkage to care, DAA treatment initiation and completion, and SVR. Data analysis was conducted using Stata Version 17.0.

### 2.4. Ethics

This study was approved by Central Adelaide Local Health Network Human Research Ethics Committee (HREC13046).

## 3. Results

A total of 230 clients received a point-of-care hepatitis antibody C test at the Hutt St homelessness service (Table 1). Of these, four in five were male (n = 182, 79%), and one in four identified as Aboriginal and/or Torres Strait Islander (n = 65, 28%). The average age was 44 years (SD 11.4, range 18–82), with one in three clients aged older than 50 years (n = 75, 33%). More than half the clients who received a point-of-care hepatitis antibody C test reported previously receiving an antibody test (n = 130, 57%) (Table 1). More than eight in ten (n = 194, 84%) clients answered that it was important for them to receive their results on the same day as their tests, and more than nine in ten (n = 215, 93%) answered that they would prefer to have the finger prick point-of-care test. The median (IQR) interval from testing until treatment was 103 (55–311) days.

### HCV Point-of-Care Testing and Treatment Outcomes

**Figure 1:**
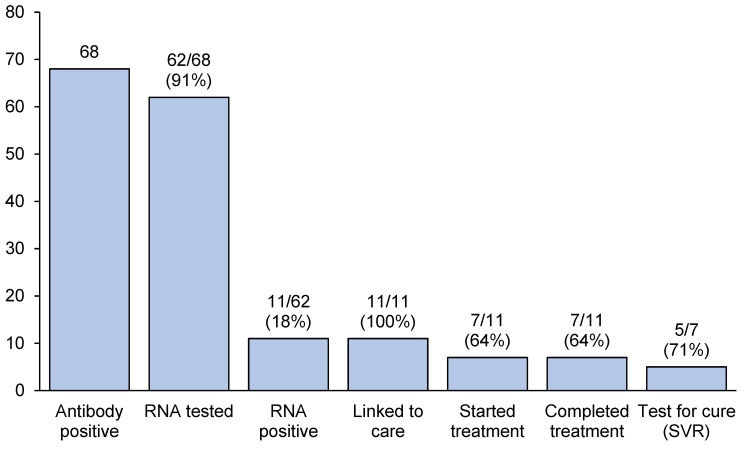
Hepatitis C cascade of care among people who received a positive hepatitis C antibody point-of-care test at Hutt St homelessness service, (N = 68).

Among the 230 clients who received a point-of-care HCV antibody test, approximately one in three (30%, 68/230) were positive (Figure 1). Among those who tested HCV antibody positive, almost all successfully received a subsequent point-of-care HCV RNA test (91%, 62/68). Among the six clients who did not successfully receive an HCV RNA test result, the test failed during operation and the clients were not available or willing to be retested. Of those who received a point-of-care HCV RNA test, 11 clients tested HCV RNA positive (18%, 11/62), all of whom were successfully linked to treatment (100%, 11/11). Notably, of the 11 clients who tested HCV RNA positive, 3 clients had received prior treatment. Among the 11 clients who were linked to care, approximately two-thirds completed treatment (64%, 7/11). Five clients (71%, 5/7) received an SVR test for cure, all of whom achieved viral clearance.

## 4. Discussion

In this study, we evaluated a nurse and peer-led HCV model of care which established testing and treatment pathways using point-of-care testing at a homelessness service in Adelaide, Australia. A total of 230 clients received HCV testing within a six-month time interval, of whom 68 (30%) received a positive antibody test and 11 (5%) received a positive RNA test. Among those who tested HCV RNA positive, around two-thirds started and completed a course of DAA treatment (64%). While HCV RNA prevalence was lower in our study than other Australian studies implemented in homeless services, a higher proportion of clients started treatment and achieved cure [10,11]. The acceptability of HCV point-of-care testing was high, with almost all clients reporting that they prioritise same-day test results, and preference point-of-care testing over standard venepuncture. The findings from this study highlight how HCV point-of-care testing can be used to establish testing and treatment pathways in priority settings with limited clinical capacity.

Whilst this project offered point-of-care testing as an alternative to venous blood draws, it still followed conventional HCV testing approaches by utilising both antibody and RNA testing. Other recent HCV models of care have opted to further streamline the testing pathway by exclusively offering HCV RNA tests, thereby reducing the risk of loss-to-follow up [12]. However, the cost of HCV RNA testing far exceeds that of antibody testing (AUD 60 vs. AUD 10), increasing the overall costs of this HCV model of care. Furthermore, the time to obtain a result is longer (60 min vs. 5–20 min). Therefore, there remains some uncertainty about when it is most appropriate to offer HCV antibody or RNA testing as the first step in the cascade of care [12]. We observed that all participants were willing to wait on-site during the 20 min read time for the HCV antibody test result. This time period provided valuable engagement and education opportunities which potentially led to very high retention of participants reflexing directly to RNA testing following a positive antibody test. Starting the cascade with the longer 60 min RNA test may lead to participants being unwilling to wait for the result; thus, the engagement opportunity can be missed. In our study, more than nine in ten (91%) clients who received a positive HCV antibody test successfully received a subsequent HCV RNA test. Given the high costs of RNA testing relative to antibody testing, the findings from this study indicate that offering initial antibody testing is viable and cost-effective, particularly when chronic HCV prevalence is low.

This project successfully implemented a nurse and peer-led HCV model of care using point-of-care testing at a homelessness service. Whilst one in three clients who participated tested HCV antibody positive, less than 5% were diagnosed with a chronic infection. This finding likely reflects both the decision to not employ a risk-based testing strategy, and the progress that Australia has made towards HCV elimination. Further, one in three clients who received a chronic HCV diagnosis did not initiate DAA therapy, indicating gaps in the cascade of care. The findings from this study suggest that point-of-care testing is an effective strategy for establishing HCV testing pathways in priority settings; however, clients are likely to require enhanced support when accessing and throughout treatment.

## Figures and Tables

**Table 1:** Characteristics of clients who received an HCV antibody point-of-care test at Hutt St homelessness service and acceptability of point-of-care testing, N = 230.

Characteristic	n (%)
Sex	
Male	182 (79)
Female	46 (20)
Not recorded	2 (1)
Age group (years)	
18–29	21 (9)
30–39	58 (25)
40–49	69 (30)
50+	75 (33)
Not recorded	1 (0.4)
Aboriginal or Torres Strait Islander	
Yes	65 (28)
No	165 (72)
Not recorded	0 (0)
Ever previously HCV tested	
Yes	130 (57)
No	69 (30)
Unsure	29 (13)
Not recorded	2 (1)
Is it important for you to get your hepatitis C result on the same days as getting tested?	
Yes	194 (84%)
No	33 (14%)
Unsure	0 (0%)
Not reported	6 (3%)
If you could choose which type of testing for hepatitis C would you prefer:	
Blood taken from my vein with results available in 1 week?	9 (4%)
Finger prick test with results available same day?	215 (93%)
Not reported	3 (1%)
HCV: hepatitis c virus	

## Data Availability

Data are contained within the article.
